# Performance map of a cluster detection test using extended power

**DOI:** 10.1186/1476-072X-12-47

**Published:** 2013-10-25

**Authors:** Aline Guttmann, Lemlih Ouchchane, Xinran Li, Isabelle Perthus, Jean Gaudart, Jacques Demongeot, Jean-Yves Boire

**Affiliations:** 1 Department of Biostatistics, Medical Informatics and Communication Technologies, Clermont University Hospital, Clermont-Ferrand F-63000, France; 2 ISIT, UMR CNRS UDA 6284, Auvergne University, Clermont-Ferrand F-63001, France; 3 PEPRADE, EA 4681, Clermont-Ferrand F-63000, France; 4 SESSTIM, UMR 912 INSERM IRD AMU, Aix-Marseille University, Marseille F-13005, France; 5 Biostatistics Unit, Assistance Publique Hôpitaux de Marseille, Marseille F-13005, France; 6 AGIM, FRE CNRS 3405, J. Fourier University, La Tronche University School of Medicine, Grenoble F-38700, France

**Keywords:** Cluster detection test, Performance map, Extended power, Simulation study

## Abstract

**Background:**

Conventional power studies possess limited ability to assess the performance of cluster detection tests. In particular, they cannot evaluate the accuracy of the cluster location, which is essential in such assessments. Furthermore, they usually estimate power for one or a few particular alternative hypotheses and thus cannot assess performance over an entire region. Takahashi and Tango developed the concept of extended power that indicates both the rate of null hypothesis rejection and the accuracy of the cluster location. We propose a systematic assessment method, using here extended power, to produce a map showing the performance of cluster detection tests over an entire region.

**Methods:**

To explore the behavior of a cluster detection test on identical cluster types at any possible location, we successively applied four different spatial and epidemiological parameters. These parameters determined four cluster collections, each covering the entire study region. We simulated 1,000 datasets for each cluster and analyzed them with Kulldorff’s spatial scan statistic. From the area under the extended power curve, we constructed a map for each parameter set showing the performance of the test across the entire region.

**Results:**

Consistent with previous studies, the performance of the spatial scan statistic increased with the baseline incidence of disease, the size of the at-risk population and the strength of the cluster (i.e*.,* the relative risk). Performance was heterogeneous, however, even for very similar clusters (i.e., similar with respect to the aforementioned factors), suggesting the influence of other factors.

**Conclusions:**

The area under the extended power curve is a single measure of performance and, although needing further exploration, it is suitable to conduct a systematic spatial evaluation of performance. The performance map we propose enables epidemiologists to assess cluster detection tests across an entire study region.

## Abstract (fr)

**Résumé:**

**Contexte**

Les études de puissance ont montré leurs limites dans l’évaluation des performances des tests de détection d’agrégats. En raison de la nécessité de prendre en compte à la fois la capacité du test à rejeter l’hypothèse nulle et à localiser correctement l’agrégat, la puissance usuelle ne peut refléter la véritable performance de ces tests. De plus, ces évaluations ne traitent en général qu’un nombre limité d’hypothèses alternatives ignorant donc le comportement de ces tests sur l’ensemble d’une région d’étude. Takahashi et Tango ont proposé le concept de puissance étendue qui, au-delà de la puissance usuelle, reflète également la précision de localisation de l’agrégat. Nous proposons une méthode d’évaluation systématique, fondée ici sur la puissance étendue, pour produire une carte offrant une visualisation synoptique des performances des tests de détection d’agrégats sur l’ensemble d’une région.

**Méthodes**

De façon à explorer le comportement d’un test de détection d’agrégats sur un même type d’agrégat pour toutes les localisations possibles, nous avons fixé quatre jeux de paramètres spatiaux et épidémiologiques, de façon à simuler quatre collections d’agrégats, chacune couvrant l’ensemble de la région d’étude. Mille jeux de données ont été simulés pour chaque agrégat et soumis au scan spatial de Kulldorff. A partir de l’aire sous la courbe de puissance étendue, nous avons produit une carte de performance pour chaque jeu de paramètres.

**Résultats**

Conformément aux précédentes études, la performance du scan spatial croît avec l’incidence de base de la maladie, la taille de la population à risque et la force de l’agrégat (i.e., le risque relatif). Cependant, même pour des agrégats très similaires, la performance du test est hétérogène, suggérant l’influence potentielle d’autres facteurs.

L’aire sous la courbe de puissance étendue est une mesure unique de performance et, bien qu’elle nécessite des évaluations plus poussées, elle convient à l’évaluation spatiale systématique de la performance. La carte de performance que nous proposons autorise les épidémiologistes à évaluer les tests de détection d’agrégats sur l’ensemble d’une région d’étude.

## Background

Spatial clusters can be detected using a wide range of statistical tests [1,2], many of which are available in free software packages such as R [3,4]. Epidemiologists use local methods to detect clusters without a priori knowledge of their location, and to determine their significance. Because these cluster detection tests (CDTs) must reveal both the presence and location of clusters, performance studies have been constrained by the limitations of conventional estimation techniques. For example, a CDT may have maximum power for rejecting the null hypothesis (cluster absence), yet be incapable of accurately locating the simulated cluster. CDT performance is also a function of epidemiological and geographical context [1,5-11]. Furthermore, because epidemiological (e.g., incidence and relative risk) and geographical (e.g., spatial unit size and shape) factors tend to be intrinsically linked, their proper or common effects are difficult to evaluate. When evaluating the behavior of these CDTs in a particular region, limited knowledge can consequently be gleaned by simulating one or a few clusters in that region, and even less knowledge can be accrued from studies on other region.

Takahashi and Tango have proposed the concept of extended power (EP) [12,13] as a more accurate measure of CDT performance. This measure assesses both the probability that the null hypothesis is rejected and the accuracy of the cluster location. As such, it overcomes the inadequacy of conventional power measures. However, EP cannot eliminate the need to define what is meant by “an accurate” or “sufficiently accurate” location. The level of spatial accuracy depends upon context; for instance, an epidemiologist will require higher spatial accuracy for an ad hoc study than for a survey system. Takahashi and Tango therefore introduced a quantitative indicator of spatial accuracy, and summarized CDT performance using an EP curve in conjunction with this spatial accuracy indicator.

In this work, we propose a method that integrates the area under the EP curve (AUC_EP_) in order to produce maps that provide a global overview of CDT performance over an entire study region.

## Methods

### Clustering model

To explore CDT behavior on same-class clusters in all possible locations, we set common spatial and epidemiological characteristics for four cluster collections covering the entire study region. The study region was the Auvergne region (France), divided into *n* = 221 spatial units (SUs) equivalent to U.S. ZIP codes. The exhaustive collection of approximately circular clusters with four SUs was identified within the study region. To achieve this outcome, the 221 SUs were successively associated with their three nearest neighbors as defined by Euclidian distances between the SU centroids. To obtain four cluster collections, we applied four combinations of two baseline risks (incidences) and two relative risks to the same at-risk population, whose size was estimated by mean annual number of live births.

For a realistic analysis, we used data archived in CEMC (birth defects registry for the Auvergne region) and INSEE (National Institute of Statistics and Economic Studies) databases. We collected two categories of data from 1999 to 2006: all birth defects and cardiovascular birth defects. Both datasets were sorted by SU. The number of live births was approximated by the number of birth declarations in the at-risk population. Global annual incidences of all birth defects (I_all_) and cardiovascular birth defects (I_CV_) were estimated as 2.26% and 0.48% of births, respectively. In the analysis, we constructed risk combinations of these two incidences at relative risks of 3 and 6.

### Datasets

For each cluster within the four categories (221 × 4), we generated 1,000 datasets, i.e., a total of 884,000 datasets. Each dataset consisted of 221 rows and 5 columns. The rows contained SU coordinates (longitude and latitude), observed number of cases, size of the at-risk population (i.e., the number of live births) and expected number of cases in the specified SU. This last quantity was the product of the global incidence (I_all_ or I_CV_) and the at-risk population size in the SU. The observed case numbers were assumed as independent Poisson variables such that

$$\left\{\begin{array}{l} \;H_{0}:E\left(N_{i}\right)=\epsilon_{i},N_{i}∼\mathrm{Pois}\left(\epsilon_{i}\right),i=1,\ldots ,n\\ H_{1}:E\left(N_{i}\right)=\pi_{i},N_{i}∼\mathrm{Pois}\left(\pi_{i}\right),\pi_{i}=I\theta \epsilon_{i}+\epsilon_{i}\left(1-I\right),\;i=1,\ldots ,n \end{array}\right)\;$$

where *N*_*i*_ is the observed number of cases, *ϵ*_*i*_ denote the expected number of cases in the *i*th SU under the null hypothesis of risk homogeneity (H_0_) and *π*_*i*_ the expected number of cases in the *i*th SU under the alternative hypothesis of one simulated cluster (H_1_). *θ* is the relative risk, and  is a binary indicator set to 1 if the *i*th SU is within the simulated cluster, and 0 otherwise.

### Measure of performance

The extended power was proposed by Takahashi and Tango as an improved measure of CDT performance. For a particular cluster, global performance is the weighted cumulative sum of the contribution of each detected cluster in all submitted datasets. Here, we summarize the construction of the performance indicator. For a more detailed description, the reader is referred to Takahashi and Tango [12,13].

Within a simulated cluster of *s* SUs, if the null hypothesis is rejected, the size *l* of a detected cluster and its s* SUs (where s* denotes a subset of *s*) are recorded. A maximum cluster size *L* is imposed, such that if *l > L*, the detected cluster is discarded. This limit prevents very large, meaningless clusters from contributing to CDT global performance. In this work, *L* was set to 30 SUs.

All eligible detected clusters (EDCs), i.e. with *l* ≤ *L*, are counted and sorted by *l* and s*. For each combined value of *l* and s*, the proportion of corresponding detected clusters (*P*_(l,s*)_*)* in all submitted datasets is assigned a weight *W*_(l,s*)_. This weight is also a function of the detection accuracy (i.e., the correct location of the simulated cluster). Thus, Takahashi and Tango define *W*_(l,s*,w+,w−)_ as

$$W_{\left(l,s^{*},w^{+},w^{-}\right)}=\sqrt[{}]{\left[1-\min\left\{w^{-}\left(s-s^{*}\right),1\right\}\right]\left[1-\min\left\{w^{+}\left(l-s^{*}\right),1\right\}\right]}$$

where *w*^*−*^ and *w*^*+*^ are penalties for false negative and false positive SUs, respectively. The penalties *w*^*−*^ and *w*^*+*^ are determined according to the following constraints. For *w*^*−*^, detected clusters that generate no false negative must fully contribute to global performance, and those that induce *s* false negatives must be discarded. These constraints are satisfied when

$$w^{-}=1/s$$

For *w*^*+*^, detected clusters that generate no false positive must fully contribute to global performance, and those that induce at least *l*_*0*_ false positives must be discarded. These constraints are satisfied when

$$w^{+}=1/l_{0}$$

So that *l*_*0*_ is not assigned arbitrarily, Takahashi and Tango specify the ratio

$$q=w^{+}/w^{-}$$

To favor sensitivity over specificity (as is usually preferred), *w*^*−*^ is greater than or equal to *w*^*+*^; thus *l*_*0*_ *≥ s* because *1/s ≥ 1/l*_*0*_. For example, when:

•

$$l_{0}=s,w^{-}=w^{+}\;\mathrm{and}\;q=1;$$

•

$$l_{0}=2s,w^{-}=2w^{+}\;\mathrm{and}\;q=0.5;$$

•

$$l_{0}\to \infty ,w^{+}=0\;\mathrm{and}\;q=0\text{.}$$

For each value of *q*, the extended power is the cumulative sum of *W*_(l,s*,q)_ *× P*_(l,s*)_, where *l* runs from 1 to *L* and s* runs from 0 to *s*. CDT global performance in detecting a particular cluster is then represented by the extended power curve with *q* running from 0 to 1. At any point on this curve, the extended power is, by construction, between 0 and 1. Furthermore, we note that the extended power is a monotonically decreasing function of *q*. Consequently, the area under the extended power curve (AUC_EP_), defined by

$$\mathrm{AU}C_{\mathrm{EP}}=\int_{q=0}^{1}\left(W{\;}_{\left(l,s^{*},q\right)}\times P_{\left(l,s^{*}\right)}\right)\mathrm{dq}$$

is between 0 and 1, with 0 signifying an inoperative CDT (s^*^ always null) and 1 a perfect CDT (H_0_ always rejected, with all detected clusters exactly overlaying the simulated cluster). As suggested by Takahashi and Tango [13], we used the area under the extended power curve as the measure of CDT performance.

### Performance mapping

Global performance was visualized over the entire region using maps representing the measured AUC_EP_ for each collection of clusters.

The AUC_EP_ is a measure of a cluster and thus associated with four SUs. In order to obtain a global overview on a single map, we assigned the AUC_EP_ value of each cluster, to its central SU. Thus, we affected a single measure of AUC_EP_ to each SU of the map. As we defined four cluster collections for four risks combination (incidence and relative risks), we produced four performance maps.

### Kulldorff’s Spatial scan statistic

In this study, we selected Kulldorff’s spatial scan statistic [14,15], a well-known and widely used CDT whose performance has been studied by many authors [1,6,10,16]. The spatial scan statistic detects the most likely cluster based on locally observed statistics of likelihood ratio tests. The scan statistic considers all possible zones *z* defined by two parameters: a center that is successively placed on the centroid of each SU, and a radius varying between 0 and a predefined maximum. The true geography being delineated by administrative tracts, i.e., each zone *z* defined by all SUs whose centroids lie within the circle, is irregularly shaped. Let *N*_*z*_ and *n*_*z*_ be the size of the at-risk population and the number of cases counted in zone *z* (over the entire region, these quantities are the total population size *N* and the total number of cases *n*, respectively). The probabilities that an at-risk case lies inside or outside zone z are respectively defined by *p*_*z*_ *= n*_*z*_*/N*_*z*_ and *q*_*z*_ *= (n − n*_*z*_*)/(N − N*_*z*_*)*. Given the null hypothesis H_0_: *p*_*z*_ *= q*_*z*_ versus the alternative H_1_: *p*_*z*_ *> q*_*z*_ and assuming a Poisson distribution of cases, Kulldorff defined the likelihood ratio statistics as proportional to

$${\left(\frac{n_{z}}{\lambda N_{z}}\right)}^{n_{z}}{\left(\frac{n-n_{z}}{\lambda \left(N-N_{z}\right)}\right)}^{n-n_{z}}I\left[n_{z}>\lambda N_{z}\right]$$

where *λ* is global incidence, and the indicator function *I* equals 1 when the number of observed cases in zone *z* exceeds the expected number under H_0_, and 0 otherwise. The circle yielding the highest likelihood ratio is identified as the most likely cluster. The *p*-value is obtained by Monte Carlo inference.

### Software

Data simulation and analysis (see Data and Script in the Additional files 1 and 2) were performed in R 2.14.0 [3,17-19] using AUVERGRID [20].

## Results

**Figure 1:**
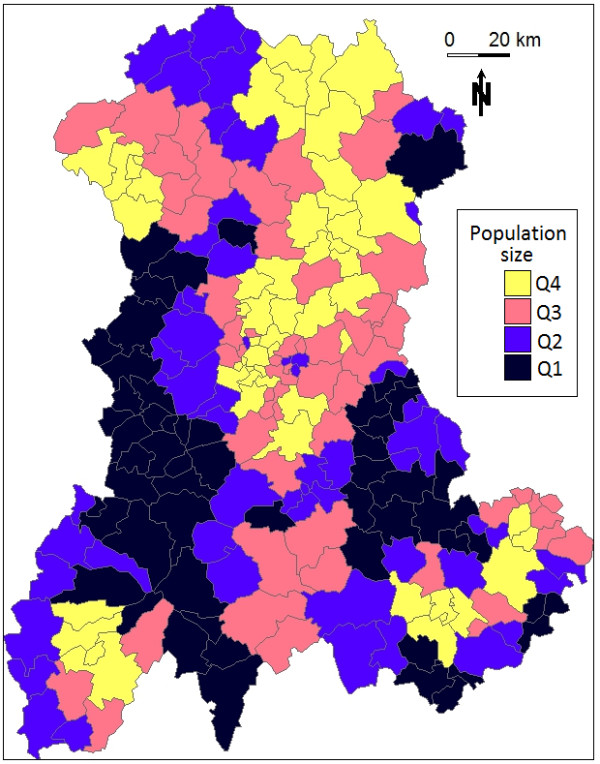
**Size of the at-risk population for each cluster in the Auvergne region, as defined by mean number of live births per year between 1999 and 2006 (source: INSEE).** Q1: ≤ 102; Q2: > 102 and ≤ 175; Q3: > 175 and ≤ 293; Q4: >293.

The Auvergne region is characterized by low and medium mountains situated around a central plain. The at-risk population (see Methods) was heterogeneously distributed throughout sparsely populated areas (mainly borderland and mountainous) and highly populated urban areas. Figure 1 shows the size of the at-risk population in each cluster, which was assigned to its central SU.

**Figure 2:**
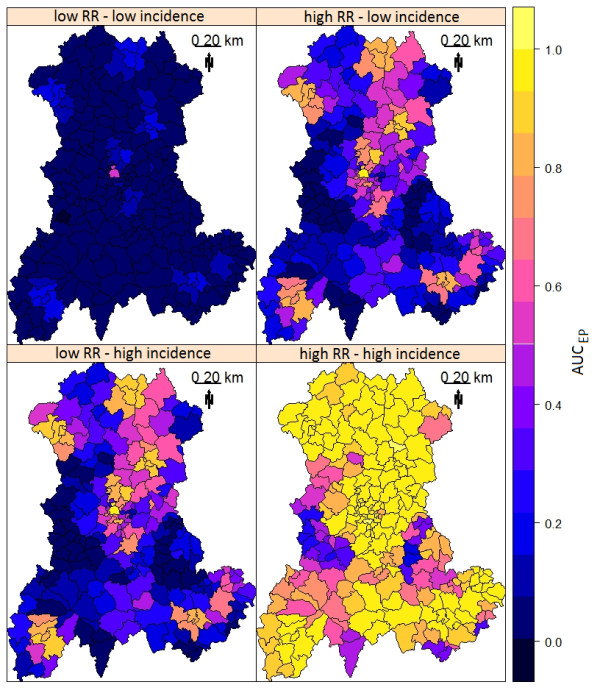
**AUC**_**EP **_**of Kulldorff’s spatial scan.** AUC_EP_ was measured for four combinations of two relative risk (RR) and two annual incidence of birth defects: low RR = 3 and high RR = 6; low incidence = 0.48% births and high incidence = 2.26% births.

**Figure 3:**
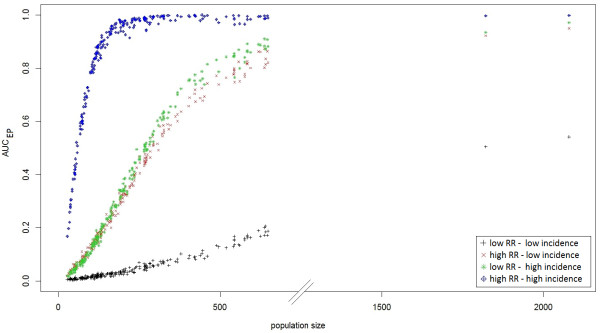
**AUC**_**EP **_**of Kulldorff’s spatial scan based on the size of the at-risk population for four combinations of two relative risk (RR) and two annual incidence of birth defects: low RR = 3 and high RR = 6; low incidence = 0.48% births and high incidence = 2.26% births.**

Figure 2 demonstrates how CDT performance improved with increasing risk level. Clearly, the CDT could not detect clusters within regions with low number of births. For these clusters, performance only marginally improved, even at the highest risk combination (Figure 3).

**Figure 4:**
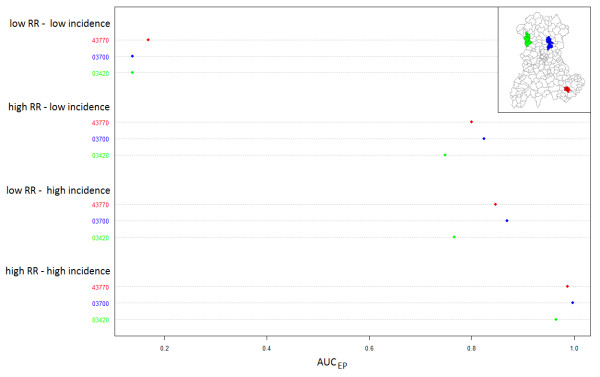
**AUC**_**EP **_**of Kulldorff’s spatial scan and locations of three simulated clusters for four combinations of two relative risk (RR) and two annual incidence of birth defects: low RR = 3 and high RR = 6; low incidence = 0.48% births and high incidence = 2.26% births.**

CDT performance increased monotonically with the at-risk population size (Figure 3). We noted a stronger heterogeneity of CDT performance for the clusters with the largest populations, especially at intermediate risk levels (Figure 3); by this, we mean that clusters with nearly the same population size led to slightly different test performance behaviors. For example, Figure 4 shows test performance in detecting three clusters centered on SUs “43770” (red cluster in the figure), “03700” (blue cluster) and “03420” (green cluster), which had population sizes of 544, 558 and 545 births (mean number over 8 years), respectively. At the lowest risk level, the red cluster was the only one even marginally detected, whereas under other configurations, the blue cluster was best detected. The worst detection performance was exhibited with respect to the green cluster, particularly at intermediate risk levels. We note that the green cluster was the only borderland cluster.

**Figure 5:**
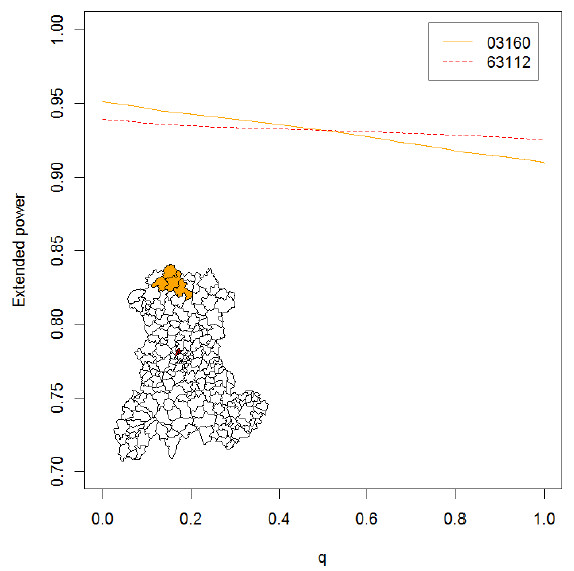
**Extended power curves for two simulated clusters.** Line 03160: cluster centered on the SU with zip code 03160 (northwest Auvergne); line 63112: cluster centered on the SU with zip code 63112 (central Auvergne). Both clusters were simulated with a relative risk of 6 and a baseline incidence of birth defects set to 2.26%.

Some summary statistics of the AUC_EP_ distributions are displayed in Table 1. Figure 5 shows two different extended power curves (and thus two different CDT behaviors) that have nearly equal AUC_EP_. One of these clusters was centered on SU “03160”, the other on SU “63112”.

**Table 1:** **AUC**_**EP **_**distribution for each risk combination and category of at-risk population size**

**Risk combination**	**Number of births**^**a**^	**AUC**_**EP**_	
		***Mean (SD)***	***Min - Max***
I_CV_ and RR = 3	≤ 102	0.010 (0.003)	0.003 - 0.020
	[102, 175]	0.021 (0.006)	0.007 - 0.033
	[175, 293]	0.043 (0.013)	0.023 - 0.077
	> 293	0.133 (0.089)	0.055 - 0.542
I_all_ and RR = 3	≤ 102	0.070 (0.028)	0.019 - 0.138
	[102, 175]	0.183 (0.038)	0.119 - 0.268
	[175, 293]	0.382 (0.075)	0.246 - 0.543
	> 293	0.713 (0.117)	0.492 - 0.950
I_CV_ and RR = 6	≤ 102	0.061 (0.025)	0.016 - 0.110
	[102, 175]	0.185 (0.047)	0.114 - 0.297
	[175, 293]	0.412 (0.083)	0.277 - 0.553
	> 293	0.768 (0.113)	0.524 - 0.971
I_all_ and RR = 6	≤ 102	0.511 (0.162)	0.168 - 0.787
	[102, 175]	0.874 (0.050)	0.783 - 0.959
	[175, 293]	0.970 (0.019)	0.915 - 0.995
	> 293	0.990 (0.010)	0.964 - 1

^a^mean number between 1999 and 2006.

Generation of one performance map from 221,000 datasets required about 5 days of computational time using the AUVERGRID grid.

## Discussion

Takahashi and Tango [13] have suggested using the AUC_EP_ to compare performance between CDTs. We used this synthetic indicator, suitable for compiling maps, to describe CDT performance. It thus fulfills our primary goal of realizing a systematic performance assessment of a CDT over an entire study area, rather than over only a few clusters. This mapping method, although using Takahashi and Tango’s extended power, is not dependent on this concept. Our method can use any other indicator that meets the requirements of being a scalar (i.e., a single measure of performance) indicating both the spatial accuracy of the detection and the capacity of cluster detection tests to reject the null hypothesis.

Interpretation of the AUC_EP_ requires further exploration, however. Although a higher AUC_EP_ clearly signifies stronger CDT performance, quite different behaviors can yield the same AUC_EP_. As shown in Figure 5, different curves can possess very similar AUC_EP_ values. This figure shows the extended power curves “03160” and “63112”, whose AUC_EP_ values are nearly equal (0.931 and 0.932, respectively), but which reflect different CDT behaviors. The procedures used to construct these curves are described in detail within separate spreadsheets (see EP curve in the Additional file 3).

The curve “63112” is nearly horizontal, indicating that the EDCs (H_0_ rejected, and cluster size *l* < maximum cluster size *L*) located the simulated cluster with high accuracy. As *q* increases, less tolerance is given to false positives until, eventually, only EDCs with at least one true positive and less than *s* false positives can contribute to the extended power. A near zero slope thus indicates that the same detected clusters, all of which contain less than *s* false positives, contribute to the extended power, regardless of *q*.

The intercept of curve “63112” is 0.939, meaning that eligible clusters (*l < L*), all of which contribute to the extended power (i.e., all clusters contain at least one true positive), were detected in 93.9% of the tests (H_0_ rejected).

To summarize curve “63112”, the simulated cluster was not always detected (no H_0_ rejection or EDC without true positive); however, provided that an EDC identified at least one true positive, the location was accurate (i.e*.*, less than *s* false positives existed in the cluster).

In contrast, the curve “03160” yields the same AUC_EP_, but is negatively sloped with an intercept of 0.951. Thus, the associated CDT produced more EDCs containing at least one true positive. The negative slope indicates that a higher proportion of these EDCs generated at least *s* false positives.

To summarize curve “03160”, the test rejected H_0_ more often and/or produced more EDCs, but located the simulated cluster with less accuracy (i.e., this analysis produced more than *s* false positives).

One particular curve has intercept equal to 1 (*q = 0*) and a zero slope. An intercept equal to 1 implies that the CDT always rejects H_0_ and that no false negatives exist in the EDCs. All detected clusters entirely overlap the simulated cluster, as in all other cases the weighting function *W*_(l, s*, q=0)_ is less than one. In addition, the zero slope indicates the perfect test that always exactly locates the simulated cluster. A perfect test always rejects H_0_, and detected clusters always satisfy l = s* = s (i.e., generate no false positive or negative). The AUC_EP_ of a perfect test equals one, because in all other cases *W*_(l, s*, q)_ is less than one.

The intercept of an extended power curve can be regarded as a “quantitative” feature of CDT performance (all EDCs generating true positives contribute to the extended power), whereas the slope may be thought of as a “qualitative” feature of CDT performance, assessing location accuracy. The parameter *q* can, in fact, be regarded as a continuous indicator reflecting to what extent a detected cluster must accurately locate the simulated cluster to contribute to the performance measure.

As shown in Figure 5, however, if an entire curve is condensed into a single measure (such as the AUC), some information is lost, because CDTs with different behaviors (i.e., curves with different shapes) can yield the same performance value.

Consequently, the impact of CDT behavior on the extended power curve must be thoroughly explored, and behaviors relevant to a particular research or application need to be defined. Through such exploration, the extent to which the AUC_EP_ is a relevant performance measure, and the purposes for which it is most suited, can be determined.

The EP has the advantage of requiring only one arbitrarily set parameter. In this work, the parameter *L*, that determines the maximum allowed size for EDCs, has been set to 30 SUs. Takahashi and Tango [12] initially proposed to set the limit *L* to one fourth or one third of region size (in numbers of SUs). The authors stated that it was not unreasonable to assume that an actual cluster size will be less than such a limit. Such arguments are often open to dispute but in any case, it is an arbitrary decision. In our view, it would be more correct to set *L* according to the size *s* of the simulated cluster because, in the simulation, it is the “real” cluster. By construction, the consequences of this arbitrary setting are limited to the lowest values of *q.* Indeed, low values of *q* mean that EDCs with false positives are less penalized, and thus large clusters are allowed to contribute to EP. In our case (*L* = 30), only values of extended power for *q* ≤ 0.15 could be underestimated, and only if we consider that detected clusters more than 7.5 times larger than the simulated cluster (4 SUs) are still meaningful. At last, compared with *L* set to 30, computing AUC_EP_ with *L* equal to 221 (i.e. without an arbitrary limit) yields a difference in AUC_EP_ always less than 10^-5^ in this work.

In producing our performance map, we chose to assign the AUC_EP_ value of a single cluster of four SUs to a single SU. Because two clusters centered on neighboring SUs likely contain common SUs, and the AUC_EP_ evaluates the detection of the entire cluster, visualizing performance on a single map can only be done in two ways. On the one hand, the AUC_EP_ of a cluster can be assigned to each of its SUs, or on the other hand, it can be assigned to a single, albeit arbitrarily chosen, SU. In the first solution, as each SU has a strong probability to be associated with more than one cluster, it is then necessary to compute a summary statistic, such as the mean, to produce a single map. In our view, it seems more comprehensible to arbitrary assign the performance measure for the whole cluster on a single SU. As we simulated more or less circular clusters, the central SU of the cluster was naturally chosen for this assignment. When simulating different cluster shapes, this choice will clearly be less obvious. We nevertheless recommend assigning the performance measure to the SU where the centroid of the cluster is located.

Authors who have studied CDT behavior mentioned its dependence on epidemiological and geographical factors [1,5-11]. Consistent with previously published results, the performance of Kulldorff’s spatial scan, and more generally, all local CDTs, improves in study regions of small SUs, large populations, high incidence of the studied phenomenon and for clusters with strong relative risk. Furthermore, as shown in Figure 4 and Table 1, the variation in AUC_EP_ among very similar simulated clusters (identical length, shape, population size and risk association) suggests that other factors influence CDT performance. To our knowledge, no other simulation study has been performed to both assess and visualize CDT performance over an entire region. Until now, authors have always considered a limited set of simulated clusters with particular epidemiological or geographical characteristics of interest. Consider the typical example of population size effect. To assess this effect, clusters are generally simulated in only a few arbitrarily chosen locations where a CDT behavior is assumed to be representative of its behavior in any other “similar” location. Usually, clusters in rural areas are compared with clusters in urban areas. Such studies are not sufficient to assess this factor that, as we have shown (Figure 3), has a strong relationship with CDT performance. Furthermore, population size cannot explain in itself all the variability in CDT performance.

However, some authors [21] have assessed performance on many randomly located clusters, which is a way to take into account the effect of spatial location without assessing it. It enabled them to assess the effect of factors such as relative risk or spatial resolution without the potential confounding effect of the spatial location. Still, this approach, while accounting for this effect, cannot quantify it.

Our systematic evaluation allows us to assess exactly when heterogeneity is most important, and thus within what population size range we can expect any other potential factor to have a maximum effect. In this work, we used predefined values for incidence and clustering characteristics (relative risk, shape, size and number) to generate performance maps. Epidemiologists should use reasonable values if a priori knowledge is available for some factors. However, the proper effect of any factor on CDT performance can be studied with this systematic evaluation, provided it uses suitable measure such as the AUC_EP_.

## Conclusion

Given that CDT performance depends on geographical and epidemiological context, the performance of these methods should be explored prior to monitoring a particular phenomenon in a given region. This work enables epidemiologists to study global CDT performance over an entire region. Furthermore, from a research viewpoint, our method seems beneficial for unraveling the proper effect of many factors, particularly geographical ones, on CDT performance.

## Abbreviations

AUCEP: Area under the curve of extended power; CDT: Cluster detection test; EDC: Eligible detected cluster; EP: Extended power; H0: Null hypothesis; H1: Alternative hypothesis; Iall: Incidence of all birth defects; Icv: Incidence of cardiovascular birth defects; RR: Relative risk.

## Competing interests

The authors declare that they have no competing interests.

## Authors’ contributions

AG and LO conceived the design, performed the study and drafted the manuscript. AG was responsible for statistical programming and data analysis. JD, JG, IP, XL and JYB contributed to manuscript revision. All authors read and approved the final manuscript.

## Supplementary Material

Additional file 1 Script: This file is an r script (script.r) containing a complete procedure to define the collection of clusters, simulate the datasets, perform the test and plot the corresponding performance map. Click here for file

Additional file 2 Data: This is a zip file (Data.zip) containing the population data in an r format (Pop.rda) and a folder with the shapefiles for the Auvergne region. Click here for file

Additional file 3 **EP curve: This file is an Excel spreadsheet (EP curve.xls) containing two worksheets.** Sheets “03160” and “63112” describe step-by-step construction of EP curves for clusters centered on SU “03160” and SU “63112”, respectively. In both constructions, the relative risk is set to 6 and the baseline incidence of birth defects is assumed to be 2.26%. To toggle between the corresponding procedures for calculating EP, the user need only alter the value of *q* in cell D41. Click here for file
